# Supplementary material for: Content-rich biological network constructed by mining PubMed abstracts
Source: BMC Bioinformatics. 2004 Oct 8;5:147. doi: 10.1186/1471-2105-5-147 (PMC528731; doi:10.1186/1471-2105-5-147)
Supplement: Additional File 2 — The original results of the above study (non-essential files are deleted to keep the file size under the limit set by BMC bioinformatics). [file 1471-2105-5-147-S2.bz2 › chilibotAdditionalFile2/dip20/665ID10428814E11059/html/TNFRSF5_TRAF3.html]

 


 **TNFRSF5** and **TRAF3** 
  
Found 115 abstracts in PubMed, retrieved 20.  
 

 What does Google say? 
 PDF only 
| .edu only 

---

**Interactive relationship** (e.g. stimulation, inhibition, etc)

**Neutral relationship**- Downstream regulator TANK binds to the CD40  [ **TNFRSF5** ]  recognition site on  **TRAF3** .  Ref: 12005438 Structure Camb , 2002
- TANK and CD40  [ **TNFRSF5** ]  bind to the same crevice on  **TRAF3** .  Ref: 12005438 Structure Camb , 2002
- LMP 1  [ **TRAF3** ]  also differs from CD40  [ **TNFRSF5** ]  in its apparent independence of a ligand for its signaling.  Ref: 12388716 J Virol, 2002
- LMP 1  [ **TRAF3** ]  differs dramatically in its structure from CD40  [ **TNFRSF5** ] .  Ref: 12388716 J Virol, 2002
- CD40  [ **TNFRSF5** ]  ligation downregulates EBNA 2 and LMP 1  [ **TRAF3** ]  expression in EBV transformed lymphoblastoid cell lines.  Ref: 12115504 Int J Cancer, 2002
- LMP 1  [ **TRAF3** ]  has six membrane spanning domains as opposed to CD40  [ **TNFRSF5** ]  s one.  Ref: 12388716 J Virol, 2002
- CD40  [ **TNFRSF5** ]  and LMP1  [ **TRAF3** ]  bind similar sets of cellular signalling proteins and activate overlapping signalling pathways.  Ref: 12401515 Cell Signal, 2003
- The data presented here show that the shared binding site for TRAF2 and  **TRAF3**  accounts for receptor internalization, and the majority of signaling through CD40  [ **TNFRSF5** ] , but is redundant with the TRAF6 binding site for activation of p38 and NFkappaB signaling pathways.  Ref: 11754002 Eur J Immunol, 2002
- Latent membrane protein 1 LMP1  [ **TRAF3** ]  is an Epstein Barr virus EBV encoded, ligand independent receptor that mimics CD40  [ **TNFRSF5** ] .  Ref: 12805217 EMBO J, 2003
- Latent membrane protein 1 LMP1  [ **TRAF3** ] , a protein encoded by Epstein Barr Virus EBV, appears to mimic CD40  [ **TNFRSF5** ]  in multiple ways.  Ref: 12401515 Cell Signal, 2003
- CD40  [ **TNFRSF5** ]  crosslinking led to a partial downregulation of EBNA 2, EBNA3 6 and LMP 1  [ **TRAF3** ]  in LCLs, paralleled by downregulation of Cp promoter activity.  Ref: 12115504 Int J Cancer, 2002
- Antisense oligodeoxynucleotide ODN for  **TRAF3**  inhibited CD40  [ **TNFRSF5** ]  induced activation of MEK1 ERK pathway by decreasing expression of  **TRAF3**  protein, but antisense ODNs for TRAF, and 6 were ineffective.  Ref: 12220533 Arch Biochem Biophys, 2002
- Furthermore, CD40  [ **TNFRSF5** ]  mediated enhancement of IL 4 driven germline C epsilon transcription was inhibited by antisense ODN for  **TRAF3**  and by a MEK1 inhibitor, PD98059.  Ref: 12220533 Arch Biochem Biophys, 2002
- In these cases the Hodgkin and Reed Sternberg HRS cells express the EBV encoded proteins LMP1  [ **TRAF3** ]  and LMP2, which can mimic CD40  [ **TNFRSF5** ]  and the B cell receptor, respectively, and therefore may play a critical role in facilitating the survival of HRS cells.  Ref: 12078898 Ann Oncol, 2002

**Non-interactive relationship** (e.g. studied together, co-existance, homology, etc.)

- We report here that CD40  [ **TNFRSF5** ]  induced  **TRAF3**  dependent activation of mitogen activated protein kinase MAPK extracellular signal regulated kinase ERK kinase 1 MEK1 is involved in the upregulation of IL 4 driven germline C epsilon transcription in a human Burkitt s lymphoma B cell line, DG75.  Ref: 12220533 Arch Biochem Biophys, 2002
- Additionally, fusion constructs of the transmembrane domain of LMP1  [ **TRAF3** ]  and the intracellular signaling domain of CD40  [ **TNFRSF5** ] , TNF R1, and TNF R2 likewise transactivated the CD83 promoter via NF kappaB.  Ref: 12857898 J VirolJ Virol, 2002
- These results suggest that in DG75 cells,  **TRAF3**  induced MEK1 activation may be involved in CD40  [ **TNFRSF5** ]  mediated upregulation of IL 4 driven germline C epsilon transcription.  Ref: 12220533 Arch Biochem Biophys, 2002
- The binding site for TRAF2 and  **TRAF3**  but not for TRAF6 is essential for CD40  [ **TNFRSF5** ]  mediated immunoglobulin class switching.  Ref: 12354380 Immunity, 2002
- we demonstrate that a fusion of LMP1  [ **TRAF3** ]  with CD40  [ **TNFRSF5** ]  recruits TRAF2 intracellularly.  Ref: 12805217 EMBO J, 2003
- The latent membrane protein 1 LMP 1  [ **TRAF3** ]  oncoprotein of Epstein Barr virus EBV is a constitutively active, CD40  [ **TNFRSF5** ]  like cell surface signaling protein essential for EBV mediated human B cell immortalization.  Ref: 12610149 J Virol, 2003
- Since CD40  [ **TNFRSF5** ]  crosslinking can reportedly inhibit the growth of EBV transformed lymphoblastoid cell lines LCLs, we have examined the effect of CD40  [ **TNFRSF5** ]  ligation on the expression of EBNAs and LMP 1  [ **TRAF3** ]  and on Cp EBV promoter activity together with several phenotypic markers.  Ref: 12115504 Int J Cancer, 2002
- We modified the phenotype of the Group I BL cells by LMP1  [ **TRAF3** ]  transfection or CD40  [ **TNFRSF5** ]  ligation.  Ref: 12115526 Int J Cancer, 2002
- The effects of resistin on expression of vascular cell adhesion molecule VCAM 1 and monocyte chemoattractant chemokine MCP 1 were studied in addition to CD40  [ **TNFRSF5** ]  receptor, CD40  [ **TNFRSF5** ]  ligand induced MCP 1 expression, and tumor necrosis factor receptor associated factor 3  **TRAF3** , an inhibitor of CD40  [ **TNFRSF5** ]  signaling.  Ref: 12874180 CirculationCirculation, 2002
- The crystal structure of a region of TANK bound to  **TRAF3**  has been determined and compared to a similar CD40  [ **TNFRSF5** ]   **TRAF3**  complex.  Ref: 12005438 Structure Camb , 2002
- The fusion of the transmembrane domain of LMP1  [ **TRAF3** ]  to signaling domains of CD40  [ **TNFRSF5** ] , TNF R1 and Fas activates their signaling.  Ref: 12805217 EMBO J, 2003
- In this review, we will compare and contrast the signalling mediated by CD40  [ **TNFRSF5** ]  and LMP1  [ **TRAF3** ] .  Ref: 12401515 Cell Signal, 2003
- These results suggest that binding to TRAF2 and or  **TRAF3**  but not TRAF6 is essential for CD40  [ **TNFRSF5** ]  isotype switching and activation in B cells.  Ref: 12354380 Immunity, 2002
- The recognition motif PxQxT is presented in a boomerang like structure in TANK that is markedly different from the hairpin loop that forms in CD40  [ **TNFRSF5** ]  upon binding to  **TRAF3** .  Ref: 12005438 Structure Camb , 2002
- Furthermore, resistin upregulates adhesion molecules and chemokines and downregulates TRAF 3  [ **TRAF3** ] , an inhibitor of CD40  [ **TNFRSF5** ]  ligand signaling.  Ref: 12874180 CirculationCirculation, 2002
- CD40  [ **TNFRSF5** ]  and its viral mimic, LMP1  [ **TRAF3** ]  similar means to different ends.  Ref: 12401515 Cell Signal, 2003
- Despite many similarities shared between CD40  [ **TNFRSF5** ]  and LMP1  [ **TRAF3** ] , they also differ substantively.  Ref: 12401515 Cell Signal, 2003
- To define the role of TRAF proteins in CD40  [ **TNFRSF5** ]  dependent isotype switching in B cells, we introduced wild type WT and mutant CD40  [ **TNFRSF5** ]  transgenes that lacked the binding motifs for TRAF6 CD40deltaTRAF6, TRAF2 and  **TRAF3**  CD40deltaTRAF2 3, or both CD40deltaTRAFs into B cells of CD40  [ **TNFRSF5** ]  mice.  Ref: 12354380 Immunity, 2002
- Like ligand activated CD40  [ **TNFRSF5** ] , LMP 1  [ **TRAF3** ]  activates NF kappaB and Jun kinase signaling pathways via binding, as a constitutive oligomer, to tumor necrosis factor receptor associated factors TRAFs.  Ref: 12610149 J Virol, 2003
- Taken together, these data further our understanding of the function of LMP1  [ **TRAF3** ]  in epithelial cells and suggest that LMP1  [ **TRAF3** ] , similar to its mammalian homolog CD40  [ **TNFRSF5** ] , can exert opposing effects on cell survival depending on the nature of the apoptosis trigger.  Ref: 12504573 Virology, 2002
- The critical role of TRAF1 in the regulation of TRAF2 dependent JNK signaling is particular to the TRAF binding domain of LMP1  [ **TRAF3** ] , since a homologous region in the cytoplasmic tail of CD40  [ **TNFRSF5** ]  or the TRADD interacting domain of LMP1  [ **TRAF3** ]  signal on the JNK axis independently of TRAF1 status.  Ref: 12502848 J Virol, 2003
- Stable transfectants expressing wild type or mutant forms of the cytoplasmic domain of CD40  [ **TNFRSF5** ]  suggested that  **TRAF3** , but not TRAF2, binding was essential for CD40  [ **TNFRSF5** ]  mediated RANTES expression.  Ref: 12122011 J Biol Chem, 2002
